# Spatial immune landscapes of SARS-CoV-2 gastrointestinal infection: macrophages contribute to local tissue inflammation and gastrointestinal symptoms

**DOI:** 10.3389/fcell.2024.1375354

**Published:** 2024-07-17

**Authors:** Shi-Ping Xian, Zhan-Yu Li, Wei Li, Peng-Fei Yang, Shen-Hao Huang, Ye Liu, Lei Tang, Jun Lai, Fa-Min Zeng, Jian-Zhong He, Yang Liu

**Affiliations:** ^1^ Department of Pathology, The Fifth Affiliated Hospital, Sun Yat-sen University, Zhuhai, Guangdong, China; ^2^ Guangdong Provincial Key Laboratory of Biomedical Imaging and Guangdong Provincial Engineering Research Center of Molecular Imaging, The Fifth Affiliated Hospital, Sun Yat-sen University, Zhuhai, China; ^3^ Department of Ophthalmology, The Fifth Affiliated Hospital, Sun Yat-sen University, Zhuhai, Guangdong, China

**Keywords:** COVID-19, gastrointestinal tract, macrophages, inflammation, immune cell

## Abstract

**Background:**

In some patients, persistent gastrointestinal symptoms like abdominal pain, nausea, and diarrhea occur as part of long COVID-19 syndrome following acute respiratory symptoms caused by SARS-CoV-2. However, the characteristics of immune cells in the gastrointestinal tract of COVID-19 patients and their association with these symptoms remain unclear.

**Methodology:**

Data were collected from 95 COVID-19 patients. Among this cohort, 11 patients who exhibited gastrointestinal symptoms and underwent gastroscopy were selected. Using imaging mass cytometry, the gastrointestinal tissues of these patients were thoroughly analyzed to identify immune cell subgroups and investigate their spatial distribution.

**Results:**

Significant acute inflammatory responses were found in the gastrointestinal tissues, particularly in the duodenum, of COVID-19 patients. These alterations included an increase in the levels of CD68^+^ macrophages and CD3^+^CD4^+^ T-cells, which was more pronounced in tissues with nucleocapsid protein (NP). The amount of CD68^+^ macrophages positively correlates with the number of CD3^+^CD4^+^ T-cells (*R* = 0.783, *p* < 0.001), additionally, spatial neighborhood analysis uncovered decreased interactions between CD68^+^ macrophages and multiple immune cells were noted in NP-positive tissues. Furthermore, weighted gene coexpression network analysis was employed to extract gene signatures related to clinical features and immune responses from the RNA-seq data derived from gastrointestinal tissues from COVID-19 patients, and we validated that the MEgreen module shown positive correlation with clinical parameter (i.e., Total bilirubin, ALT, AST) and macrophages (*R* = 0.84, *p* = 0.001), but negatively correlated with CD4^+^ T cells (*R* = −0.62, *p* = 0.004). By contrast, the MEblue module was inversely associated with macrophages and positively related with CD4^+^ T cells. Gene function enrichment analyses revealed that the MEgreen module is closely associated with biological processes such as immune response activation, signal transduction, and chemotaxis regulation, indicating its role in the gastrointestinal inflammatory response.

**Conclusion:**

The findings of this study highlight the role of specific immune cell groups in the gastrointestinal inflammatory response in COVID-19 patients. Gene coexpression network analysis further emphasized the importance of the gene modules in gastrointestinal immune responses, providing potential molecular targets for the treatment of COVID-19-related gastrointestinal symptoms.

## 1 Introduction

The enveloped RNA betacoronavirus severe acute respiratory syndrome coronavirus 2 (SARS-CoV-2) is the etiological agent causing coronavirus disease 2019 (COVID-19) and can cause severe acute respiratory syndrome. However, following the acute phase of COVID-19, patients often experience persistent or developing symptoms, including fatigue, dyspnea, cognitive impairment, and gastrointestinal (GI) disorders; this disease is known as long COVID-19 or post-COVID-19 syndrome (Joshee et al., 2022). Notably, GI symptoms, including nausea, vomiting, abdominal pain, dysgeusia/ageusia, anorexia, lack/loss of appetite, and diarrhea, persist in patients with long COVID-19. The incidence rates of long COVID-19 vary across studies and are potentially influenced by cohort size. The etiology of long COVID-19 remains unclear, and while SARS-CoV-2 primarily affects the respiratory system, evidence suggests the potential involvement of digestive tract infection (Crook et al., 2021). In the acute phase of COVID-19, high titers of SARS-CoV-2 RNA have been extracted from feces (Guan and Zhong, 2020; Lin et al., 2020; Xiao et al., 2020). In addition, SARS-CoV-2 efficiently infects and propagates in intestinal epithelial cells *in vitro*, and the virus is detectable in the stool and intestinal mucosa of some patients long after clearance from the upper respiratory tract (Alvarado et al., 2022). The above hypothesis of viral persistence in the GI system is supported by the continuous detection of residual viral antigens in the GI tissues (colon, appendix, ileum) of convalescent patients (Goh et al., 2022). Understanding the precise contribution of the GI tract to the pathobiology of long COVID-19 is beneficial for the development of potential therapeutic approaches targeting the GI tract.

Previous work from our group and other colleagues revealed GI infection caused by SARS-CoV-2 in COVID-19 patients, which expanded the virus’s infection spectrum and highlighted the importance of GI manifestations in COVID-19 patients. GI infection caused by SARS-CoV-2 was recently confirmed in nonhuman primate models (Jin et al., 2020; Xiao et al., 2020; Jiao et al., 2021). We further verified that VEGF was overproduced by enterocytes, enhancing permeability and promoting inflammation, which are related to disease severity and systemic inflammation (Vahl et al., 1996). However, while they are a vital component of the host immune response, the cellular and genomic signatures involved in GI inflammation in COVID-19 patients remain understudied.

Single-nucleus RNA sequencing (snRNA-Seq) analyses revealed substantial dysregulation of the immune response in COVID-19 autopsy tissues (Delorey et al., 2021). Multiple studies have revealed infiltrated plasma cells and lymphocytes and interstitial edema in the lamina propria in the stomach, duodenum, and rectum of COVID-19 patients (Xiao et al., 2020; Livanos et al., 2021). These pathological changes were confirmed in a nonhuman primate model, and the importance of inflammatory cytokines as determinants of pathogenesis was emphasized (Jiao et al., 2021). Increased inflammatory cytokine secretion aberrantly activates monocytes/macrophages and T-cells and increases epithelial cell migration into the bloodstream, accelerating disease progression (Mjösberg and Rao, 2018; Xiao et al., 2020). Dysfunction of intestinal macrophages impairs their role in the regulation of GI homeostasis, inflammatory responses, gut secretion, and gut motility (Yip et al., 2021). Abnormal intraepithelial lymphocytes are less able to promote cell-mediated immune responses by activating other immune cells to control viral spread, which is critical in the defense against both extracellular and intracellular pathogens (Niessl et al., 2021). Therefore, to systematically characterize the GI immune microenvironment of patients and identify key immune gene signatures, it is important to investigate stromal components, understand their significance and identify effective therapeutic targets to alleviate GI symptoms in COVID-19 patients.

The present study utilized SARS-CoV-2-infected GI tract tissue from COVID-19 patients. By conducting imaging mass cytometry, immunostaining and reanalysis of in-house transcriptome sequencing data, we delineated immune cell population variation and identified potential key signaling pathways. On the basis of these analyses, defining the interrelationships between GI mucosa damage and tissue immune cells at distinct immunopathological stages of COVID-19 will help elucidate the mechanisms of GI manifestations to better optimize COVID-19 medical management and mitigation efforts.

## 2 Materials and methods

### 2.1 Patients and specimens

Gastric, duodenal, and rectal endoscopic biopsy samples were obtained from COVID-19 patients in accordance with guidelines established by the Chinese Center for Disease Control and Prevention. All tissue specimens were formalin fixed and paraffin embedded. Written informed consent was obtained from each patient, and ethical approval was granted by the Ethics Committee of the Fifth Affiliated Hospital, Sun Yat-sen University. All the experiments adhered to the principles outlined in the Department of Health and Human Services Belmont Report and the WMA Declaration of Helsinki. Patient demographic information, sex, GI symptoms, and disease classification were extracted from the patients’ electronic medical records. Only patients whose pharyngeal swab and feces specimens tested positive for SARS-CoV-2 according to real-time reverse transcription PCR (RT–PCR) and with available follow-up and clinical data were selected for this study. Patients who had any comorbidity that affects immunological processes at the gastrointestinal level, such as tumors and autoimmune pathologies with systemic or localized effects, were excluded. The clinical information of the included patients is summarized in Table 1.

**TABLE 1:** Clinical information of the 11 COVID-19 patients.

Patient	Sex	Age	Disease classification	GI manifestation	Comorbidities
1	Male	29	Mild	Epigastric discomfort, decreased AST	Hypercholesterolemia
2	Female	37	Moderate	Anorexia, decreased ALT and AST	NA
3	Male	65	Moderate	Acid reflux, epigastric discomfort	NA
4	Female	55	Mild	Epigastric discomfort, decreased AST	NA
5	Male	79	Critical	GI bleeding, increased bilirubin, ALT, and AST	NA
6	Male	45	Mild	Diarrhea, increased bilirubin, decreased AST	NA
7	Male	32	Moderate	Nausea, vomiting, decreased AST	NA
8	Female	56	Moderate	Diarrhea	Hypertension
9	Male	28	Mild	Increased bilirubin	NA
10	Male	61	Critical	Diarrhea, increased bilirubin, ALT, and AST	Hyperthyroidism
11	Female	22	Moderate	Diarrhea, increased bilirubin, decreased AST	NA

NA: not available

### 2.2 Immunofluorescence staining

Sections were treated with 0.1% Triton X-100 in phosphate-buffered saline (PBS) for 15 min and subsequently incubated with 10% goat serum (ZSGB-BIO, ZLI-9056) for 1 h at room temperature. Subsequently, the slides were incubated overnight at 4°C with primary antibodies (anti-ACE2, Santa Cruz, Cat. No. sc390851, 1:100; anti-SARS-CoV-2 nucleocapsid, Sino Biological, Cat. No. 40143-R007, 1:500). Next, the slides were exposed to secondary antibodies (Alexa Fluor^®^ 647-conjugated goat anti-rabbit IgG, bs-0295G-AF647, Bioss, 1:100; DyLight-549 goat anti-mouse IgG secondary antibody, A23310, Abbkine, 1:100) for 1 h at room temperature, followed by three washes with PBS. Nuclei were stained with 4',6-diamidino-2-phenylindole (DAPI). Finally, the slides were imaged using a laser scanning confocal microscope (LSM880, Carl Zeiss Micro Imaging).

### 2.3 Imaging mass cytometry (IMC)

Imaging mass cytometry (IMC) staining was conducted on duodenal and stomach samples obtained from 11 COVID-19 patients following established methodology (PN00322A3). The clinical information for these samples is summarized in Table 1. In brief, 6-μm-thick formalin-fixed paraffin-embedded (FFPE) tissue sections were cut. The slides were subjected to routine dewaxing and rehydration, followed by antigen retrieval in antigen retrieval buffer (Agilent, Cat. No. S236784-2, pH 9) for 30 min in a water bath at 96°C. After cooling and rinsing with deionized water (ddH_2_O), the slides were blocked with 3% BSA in DPBS for 45 min at room temperature. Subsequently, the slides were treated with diluted metal-conjugated antibody mixtures in DPBS/0.5% BSA overnight at 4°C. Detailed information about the antibodies used in this study is provided in Table 2. The slides were then treated with a DNA intercalator (Fluidigm, Cat. No. 201192A) for 30 min at room temperature after being washed four times with DPBS/0.1% Triton X-100 (Thermo Scientific, Cat. No. 85111) and DPBS. Finally, the slides were rinsed twice in ddH2O and air-dried before IMC analysis.

**TABLE 2:** Summary of antibodies and their corresponding metal tags used for imaging mass cytometry.

Antibody	Metal	Clone	Provider	Primary antibody working dilution	Cat. No.
CD68	159Tb	KP1	Fluidigm	1/100	3159035D
Vimentin	143Nd	D21H3	Fluidigm	1/200	3143027D
CD20	161Dy	H1	Fluidigm	1/100	3161029D
CD4	156Gd	EPR6855	Fluidigm	1/100	3156033D
CD8a	162Dy	D8A8Y	Fluidigm	1/100	3162035D
CD3	170Er	Polyclonal,C-Terminal	Fluidigm	1/100	3170019D
Granzyme B	167Er	EPR20129-217	Fluidigm	1/200	3167021D
E-cadherin	158Gd	2.40E+11	Fluidigm	1/50	3158029D
p-NFkB p65 [S529]	166Er	K10-895.12.50	Fluidigm	1/50	3166026D
ACE2	173Yb	HD09SE1514-B	Sino Biological	1/100	10108-T56
NP	168Er	HA14AP3001	Sino Biological	1/200	40150-R007
Interrrcalator	191Ir/193Ir		Fluidigm	1/400	201192A

For IMC data acquisition, the tissue slides were scanned using a pulsed deep UV laser beam and simultaneously analyzed using a mass cytometer (Helios-Hyperion, Fluidigm) following the manufacturer’s instructions. An intensity map of the target proteins across each location was generated by concurrently measuring the levels of the metal isotopes linked to each region of interest.

### 2.4 IMC image visualization and neighborhood analysis

For each recorded region of interest (ROI), we used MCD Viewer (Fluidigm, version 1.0.560.6) to visualize the images and exported a series of 16-bit single-channel TIFF files. To obtain single-cell information, the 16-bit TIFF files were subsequently imported into Cell Profiler version 3.1.5. Due to scanning area limitations, 12 NP^+^ tissue samples and 4 NP^−^ tissue samples from 11 patients were selected for histoCAT analysis. Subsequently, single-cell data were normalized according to an algorithm implemented in histoCAT (version 1.76) for boxplots, spatial distribution maps, t-distributed stochastic neighbor embedding (t-SNE), and unsupervised clustering. In line with prior studies, we characterized each cluster as follows: Cluster 4 corresponds to CD8^+^ T-cells, Cluster 5 corresponds to CD4^+^ T-cells, Clusters 6 and 14 are identified as epithelial cells, Cluster 7 comprises stromal cells, Cluster 9 represents CD68^+^ macrophages, Cluster 11 contains NP-infected cells, Cluster 13 consists of CD20^+^ B-cells, and Cluster 15 is composed of active immune cells. However, Clusters 1, 2, 3, 8, 10, and 12 exhibited low expression levels, making them indistinct in terms of specific cell populations (Turnier et al., 2022). Subsequently, histoCAT was used to perform a neighborhood analysis of the immune cells from COVID-19 patients.

Neighborhood analysis was conducted according to an algorithm implemented in histoCAT (version 1.76). Cell neighbors were found by expanding three pixels. Using 999 random permutations of cell type labels and a significance threshold of *p* < 0.01, we determined whether there were meaningful connections or avoidance patterns among cell types. All the other parameters and methods used were chosen as suggested in the original publication (Schapiro et al., 2017).

### 2.5 Weighted gene coexpression network analysis

All samples were collected from the Department of Pathology, the Fifth Affiliated Hospital, Sun Yat-sen University, and gene expression information was obtained via the Illumina NovaSeq 6,000 platform. The transcriptome sequencing data are available publicly from the GSA human database under accession number HRA002115 (https://ngdc.cncb.ac.cn/gsa-human/s/geaE1XIf) (Zeng et al., 2022). We conducted weighted gene coexpression network analysis (WGCNA) to discern the key gene modules associated with GI manifestations and immune cells utilizing unsupervised clustering without predefined gene sets. The optimal soft threshold power was selected to ascertain module-trait associations, module membership, and gene significance. In brief, we initially constructed a weighted adjacency matrix based on the chosen soft threshold power. The connectedness of each gene was subsequently determined by computing its associations with other genes. The module eigengene, upon which clinicopathological features were regressed using the Limma R package, summarized the gene expression profile of each module following verification of module structure preservation through the module preservation R function. The module specific to clinicopathological features was chosen due to its highest coefficient square (r^2^) and *p*-value <0.001.

### 2.6 Gene enrichment and protein–protein interaction (PPI) network analysis

To reveal the potential biological functions and underlying mechanisms of the significantly differentially expressed genes in the MEgreen module, Gene Ontology (GO) and Kyoto Encyclopedia of Genes and Genomes (KEGG) pathway enrichment analyses were conducted using the clusterProfiler package in R software (Yu et al., 2012). GO terms, including biological processes, cellular components, molecular functions, and KEGG pathways, with an adjusted *p* < 0.05 were considered significantly enriched or depleted.

The Search Tool for the Retrieval of Interacting Genes/Proteins (STRING) database (https://www.string-db.org/), a protein association network, was utilized to identify functional interactions between the key genes in the MEgreen module. A protein–protein interaction (PPI) network was constructed using STRING, and three core networks were ultimately identified from the PPI network via molecular complex detection. As a general-purpose, open-source software platform for network biology analysis and visualization, Cytoscape (3.8.2) was used to visualize the PPI networks (Szklarczyk et al., 2019).

### 2.7 Plasma VEGF measurement

The plasma samples were collected into heparinized plastic tubes from patients with COVID-19 (n = 11). Plasma VEGF levels were measured using a Luminex Assay Human XL Cytokine Discovery Premixed Kit (R&D) based on standard techniques as previously described (Zeng et al., 2022).

### 2.8 Statistical analysis

SPSS version 21.0 software (IBM, Inc., Chicago, IL) was used for statistical analysis. Differences between groups were assessed by a *t*-test (the Mann–Whitney test). Abnormally distributed data were analyzed by a multiple-sample nonparametric test. Correlations were evaluated by Pearson correlation analysis. The experimental data were analyzed via Pearson correlation analysis, and *p* <0.05 was considered to indicate statistical significance.

## 3 Results

### 3.1 GI inflammatory response in COVID-19 patients

**FIGURE 1:**
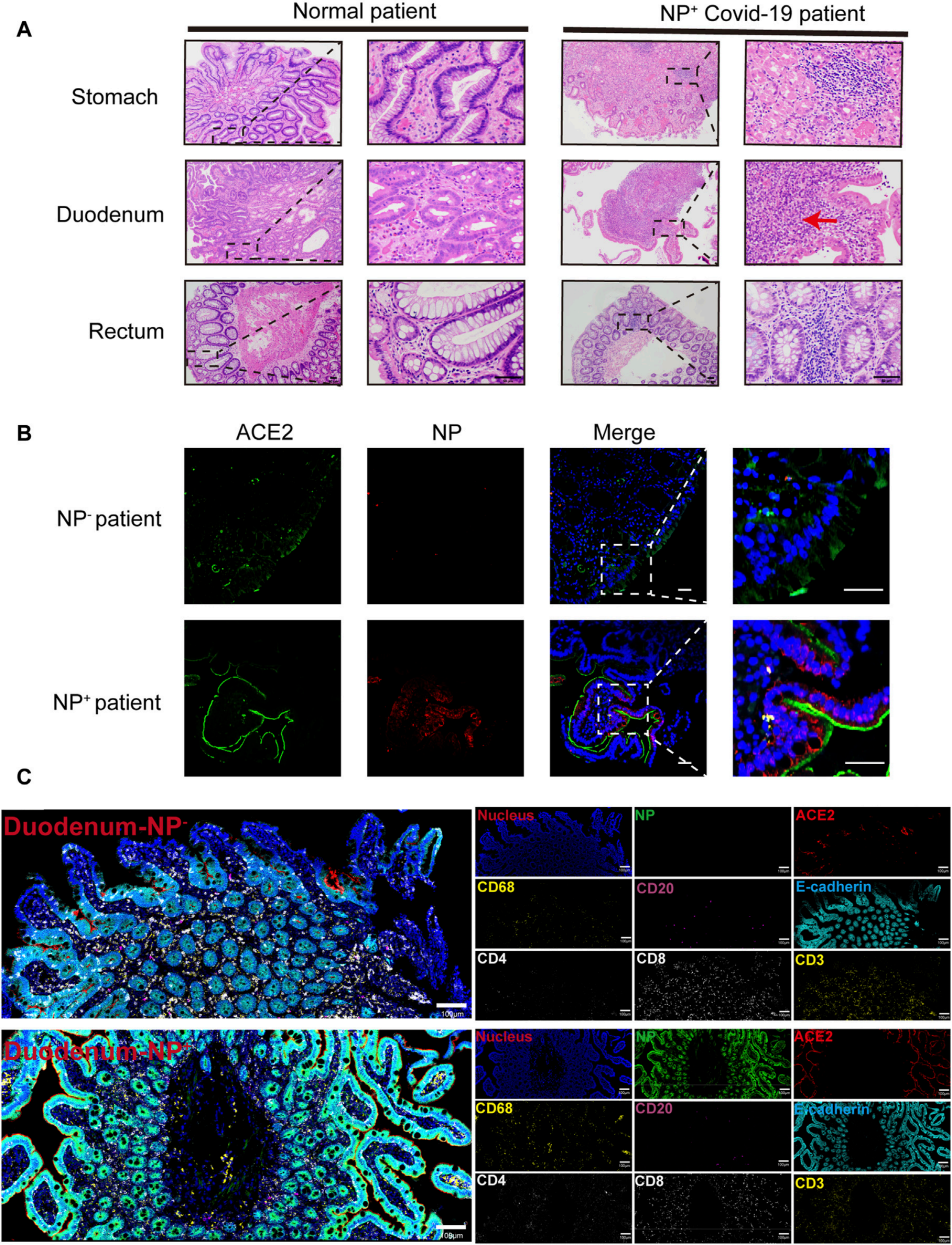
Gastrointestinal (GI) biopsies of COVID-19 patients showing histomorphologic changes. **(A)** In contrast to those of the healthy lamina propria, the gastrointestinal tracts of COVID-19 patients exhibited interstitial edema, acute inflammation (red arrow) and infiltration of plasma cells and lymphocytes (scale bar, 50 μm). **(B)** Immunofluorescence analysis of ACE2 and the SARS-CoV-2 nucleocapsid protein in the intestinal tissues of COVID-19 patients. ACE2 staining is shown in green, nucleocapsid staining is shown in red, and nuclear staining is shown in blue (scale bar, 50 μm). **(C)** Representative IMC images of gastrointestinal tissues from COVID-19 patients with (n = 12) or without (n = 4) detected NPs. Each image on the left was rendered with a selection of different markers (blue: nucleus, green: NP, red: ACE2, yellow: CD68 and CD3, magenta: CD20, cyan: E-cadherin, white: CD4 and CD8) (scale bar = 100 μm).

From February 1 to 17 April 2020, among the 95 COVID-19 patients in our cohort, 11 (11.6%) individuals presenting with GI symptoms underwent gastroscopy. As depicted in Figure 1A, the lamina propria of the GI tract, encompassing the stomach, duodenum, and rectum, in COVID-19 patients displayed dilated and congested microvasculature, interstitial edema, and plasma cell or lymphocyte infiltration, contrasting with findings in healthy individuals. Notably, acute inflammation or the appearance of a microabscess in the duodenum was observed (Figure 1A). Furthermore, immunofluorescence analysis revealed the presence of the viral nucleocapsid protein in the epithelium, suggesting that mature ACE2^+^ intestinal epithelial cells constitute the primary target cells within the intestinal epithelium (Figure 1B). As indicated in our prior investigations (Lin et al., 2020; Xiao et al., 2020), the intestinal mucosa can be infected by SARS-CoV-2, causing GI inflammatory symptoms. Given the importance of inflammation in the pathobiology of SARS-CoV-2 GI infection, we further investigated the immune cell landscape in GI tract tissues. The levels of COVID-19 microenvironment markers, i.e., CD3, CD4, CD8, CD20, CD68, vimentin, NP, ACE2, Granzyme B, E-cadherin, and phosphorylated nuclear factor kappa B (p-NFκB), were measured via IMC. We utilized IMC to generate highly multiplexed images at a 1-µm resolution. This approach was employed to identify immune cell subgroups and assess their spatial distribution within the GI tract of COVID-19 patients. As illustrated in Figure 1C, we observed CD68^+^ macrophages, CD20^+^ B-cells, and CD3^+^CD4^+^ and CD3^+^CD8^+^ T-cells in the duodenal lamina propria. Notably, the major populations in duodenal tissues that were positive for nucleocapsid protein (NP) were CD68^+^ macrophages and CD3^+^CD4^+^ T-cells, in contrast to those in NP-negative duodenal tissues. As expected, there was no significant difference in the spatial distribution of the various immune cells (Figure 1C). Similar results were observed in the stomach and rectum of COVID-19 patients (Supplementary Figures S1A–D). Histopathological examination of GI tissues from COVID-19 patients revealed pronounced acute responses, especially in duodenal tissues, characterized by the predominant presence of inflammatory cells, specifically CD68^+^ macrophages and CD3^+^CD4^+^ T-cells.

### 3.2 GI symptoms are associated with the accumulation of CD68^+^ macrophages and CD4^+^ T-cells

**FIGURE 2:**
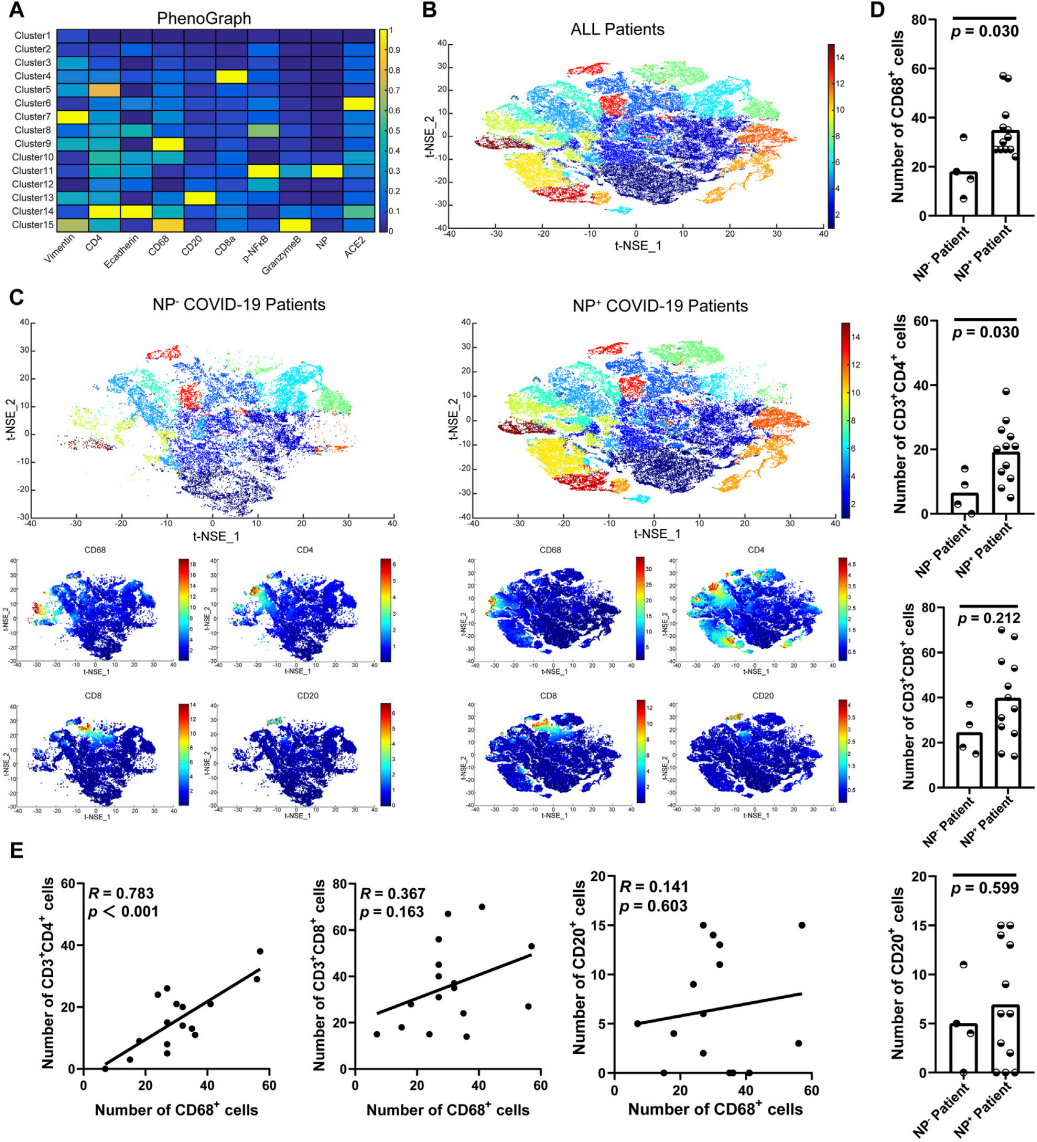
Macrophage recruitment in GI tissue from COVID-19 patients. **(A)** Cell phenotypes were visualized with a heatmap based on biomarker expression in NP^+^ tissues (n = 12) and NP^−^ tissues (n = 4). **(B)** PhenoGraph defines complex cell phenotypes based on marker expression and enables the labeling of cell phenotype clusters on a t-SNE plot. **(C)** Analysis by t-distributed stochastic neighbor embedding (t-SNE) dimensionality reduction yielded t-SNE plots of gastrointestinal tissues from COVID-19 patients with or without NP detection. Single-cell t-SNE maps showing the expression of CD68, CD4, CD8, and CD20. Plots showing NP^−^ (right) in comparison to NP^+^ (left) samples. The color spectrum on the right of the plot indicates the mean expression levels of the markers (red, high expression; blue, low expression). **(D)** Comparisons of the numbers of CD68^+^ macrophages, CD3^+^CD4^+^ T-cells, CD3^+^CD8^+^ T-cells, and CD20^+^ B-cells in the groups with or without detected NPs (Mann–Whitney test). **(E)** The scatter plots show a positive correlation between CD68^+^ macrophages and CD3^+^CD4^+^ T-cells (n = 16), but no significant correlation was observed with CD3^+^CD8^+^ T-cells or CD20^+^ B-cells (n = 16).

To further examine the accumulation of various cell types in GI tract tissues and their contribution to COVID-19 disease progression and immune responses (Jia et al., 2022; Turnier et al., 2022), the expression of cell subgroups for each patient is shown individually in Supplementary Figure S2. Patients with greater levels of NP expression demonstrated greater immune cell infiltration. By running a PhenoGraph on each sample separately to define clusters based on the 10 surface markers, we identified 15 distinct cell clusters. Among these, we definitively identified four immune cell populations, CD8^+^ T-cells (cluster 4), CD4^+^ T-cells (cluster 5), CD68^+^ macrophages (cluster 9), and B-cells (cluster 13), and then displayed all the clusters using a heatmap (Figure 2A). As shown in Figure 2A, p-NFκB was highly expressed by SARS-CoV-2 NP^+^ cells (cluster 11), and p-NFκB plays an important role in immune cell activation and cytokine secretion. All the clusters were shared across both the NP^+^ and NP^−^ subgroups, except for cluster 11, and subsequently visualized on a t-SNE map (Figure 2B, C). Immune cells, especially CD68^+^ macrophages, were more abundant in the NP^+^ group than in the NP^−^ group, suggesting a potential crucial role for these cells in COVID-19-related inflammation and GI symptoms (Figure 2C) (Lehmann et al., 2021). Quantitative analysis confirmed that duodenal tissues from the NP^+^ group exhibited greater numbers of CD68^+^ macrophages (*p* = 0.030) and CD3^+^CD4^+^ T-cells (*p* = 0.030) than did those from the NP^−^ group, while the numbers of CD20^+^ B-cells and CD3^+^CD8^+^ T-cells were essentially unchanged (Figure 2D). Correlation analysis was used to confirm the relationships among immune cells in the duodenum; CD68^+^ cell recruitment was positively associated with CD3^+^CD4^+^ T-cell, CD20^+^ B-cell and CD3^+^CD8^+^ T-cell levels in the duodenum, while only the correlation with CD3^+^CD4^+^ T-cells (*R* = 0.783, *p* < 0.001) was statistically significant (Figure 2E). The above results indicate that local intestinal inflammation may result from the interaction between CD68^+^ macrophages and CD3^+^CD4^+^ T-cell recruitment. Notably, CD68^+^ macrophages were also significantly associated with disease type, disease progression, upper gastrointestinal hemorrhage and alanine aminotransferase (ALT, Table 3), suggesting that CD68^+^ macrophages are a crucial factor in local intestinal inflammation disease progression.

**TABLE 3:** Correlations between CD68^+^ macrophages and clinicopathological features in patients with COVID-19.

	CD68^+^ macrophages	
	R	*P*
Age	0.4421	0.105
Sex	0.346	0.189
Disease classification (Nonsevere, Severe)	0.607	0.013
Disease progression	0.600	0.014
Diarrhea	−0.204	0.449
Nausea Emesis	−0.160	0.553
Acid reflux	−0.095	0.726
Epigastric discomfort	0.221	0.411
Upper gastrointestinal hemorrhage	0.586	0.017
Hepatic function impairment		
Total bilirubin (lmol/L; normal range 3.0–24.0)	0.181	0.501
ALT (U/L; normal range 7–40 in female, 9–50 in male)	0.552	0.027
AST (U/L; normal rage 13–35 in female, 15–40 in male)	−0.132	0.626
Serologic markers of disease severity		
CRP (lg/L; normal range 0.068–8.2)	0.251	0.349
D-dimers (lg/L; normal range 0–243)	0.251	0.349
Procalcitonin	0.267	0.317

*p* < 0.05 was considered to indicate statistical significance.

### 3.3 Neighborhood analysis of the inflammatory reaction of the duodenum

**FIGURE 3:**
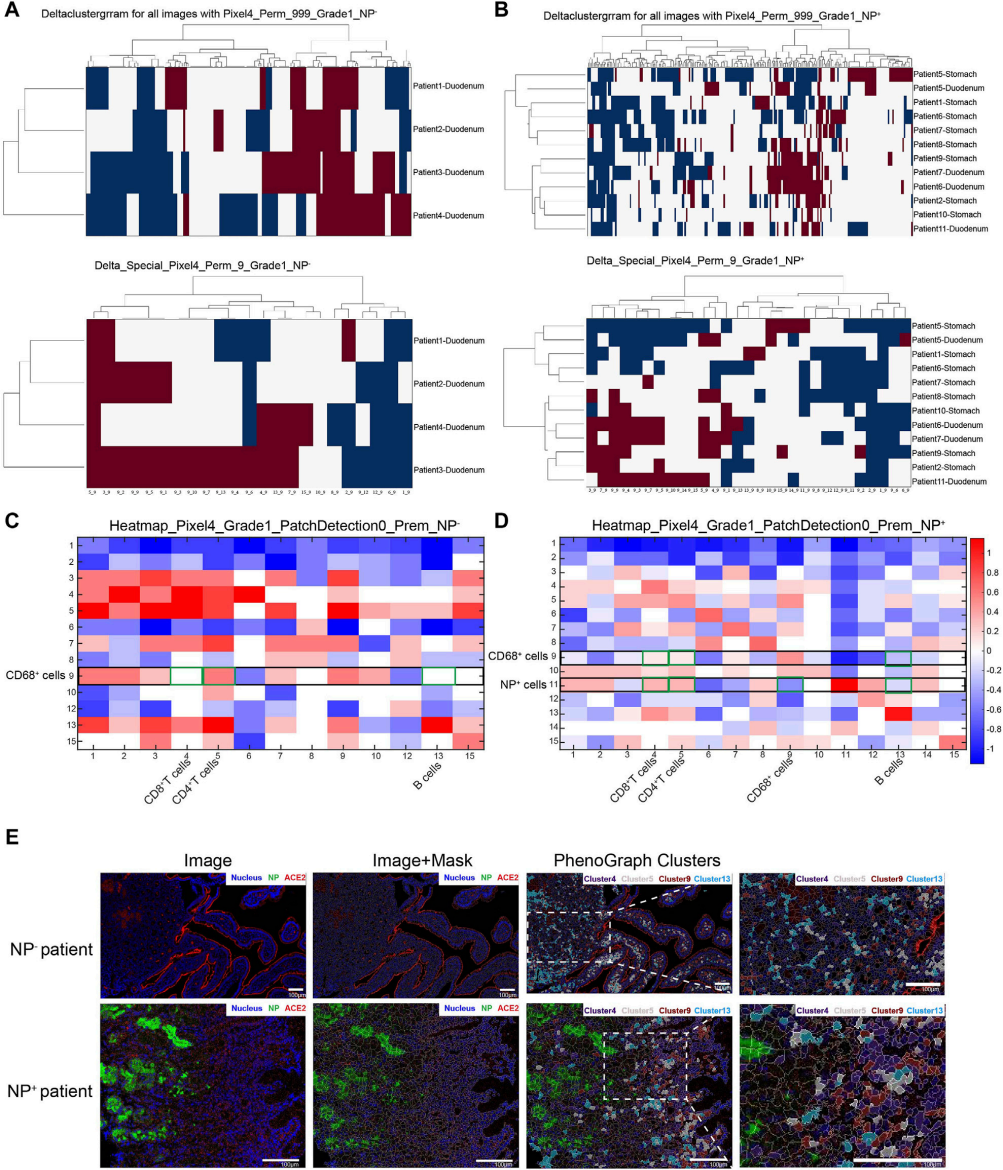
Neighborhood analysis of cell–cell interactions in the GI tract of COVID-19 patients. **(A, B)** Unsupervised neighborhood analysis visualization of all cell-to-cell interactions based on the presence of significant adjacent (red) or avoidance (blue) interactions; white denotes the absence or nonsignificance of cell contacts (permutation test, *p* < 0.01). **(C, D)** Heatmap displaying cell–cell interactions in 4 NP^−^ GI tract tissue samples **(C)** and 12 NP^+^ tissue samples **(D)** from COVID-19 patients, in which the cell type in the row significantly neighbored (red) or avoided (blue) the cell type in the column. White represents a prevalence less than 10%. **(E)** Images from the NP^−^ and NP^+^ groups are displayed in the miCAT image window using user-defined color channels (blue, nucleus; green, NP; red, ACE2). Cells of interest, such as CD8^+^ T-cells (cluster 4, purple), CD4^+^ T-cells (cluster 5, gray), CD68^+^ macrophages (cluster 9, dark red), and CD20^+^ B-cells (cluster 13, turquoise), are highlighted in the image (scale bar: 100 μm).

COVID-19-associated macrophages can drive the progression of this disease (Table 3). To explore the cell‒cell interactions among various immune cells, we applied spatial neighbor analysis to COVID-19 patients’ GI tissues via HistoCAT (Figure 3A, B). Unsupervised neighborhood analysis also revealed avoidance of the aforementioned cell clusters but robust contact between CD68^+^ macrophages and CD4^+^ T-cells in NP^−^ intestinal tissues (Figure 3C). The interaction and avoidance between CD68^+^ macrophages and other immune cell phenotypes were enriched (Figure 3B); among them, CD68^+^ macrophages (cluster 9) had significantly more interactions with multiple cell phenotypes (especially CD4^+^ T-cells, cluster 5; Figure 3C), while NP^+^ cells (cluster 11) avoided CD68^+^ macrophages (cluster 9) and CD20^+^ B-cells (cluster 13) and decreased the interaction between CD68^+^ macrophages and CD8^+^ T-cells (cluster 4) or CD4^+^ T-cells (cluster 5), suggesting that the macrophages, which act as professional antigen-presenting cells (APCs), have less interplay with T-cells at the intestinal mucosal interface, reducing their involvement in maintaining adaptive immune responses (Figure 3D, E). In contrast to CD68^+^ macrophages, intestinal NP^+^ epithelial cells were more touch with CD8^+^ or CD4^+^ T-cells (Figure 3D). Recent advances regarding an active role of intestinal epithelial cells within the mucosal immune system have revealed that they act as non-professional antigen-presenting cells (APCs), activating subsets of T-cells with regulatory function (Nakazawa et al., 2004; Roda et al., 2010). Hence, under some conditions, antigens presented by IECs cause a suppression rather than adaptive immune response.

### 3.4 Weighted gene coexpression network analysis (WGCNA)

**FIGURE 4:**
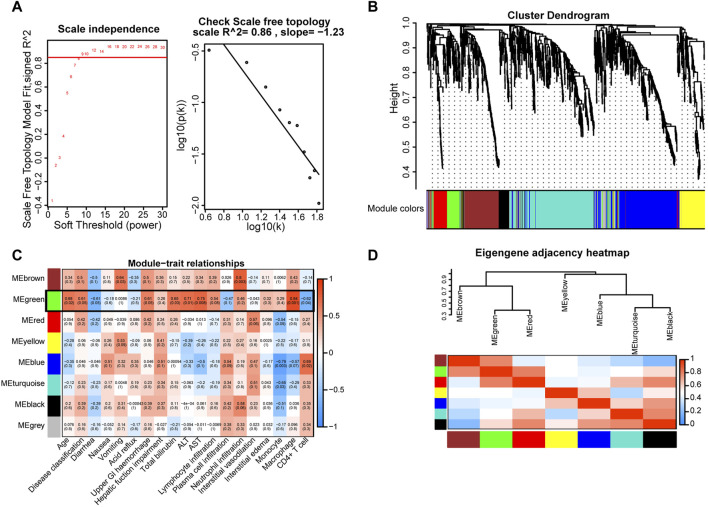
Key gene modules related to GI manifestations and immunoreactions were identified via WGCNA in COVID-19 patient tissues. **(A)** Determination of soft-thresholding power. **(B)** A dendrogram depicting the clustering, based on dissimilarity measures (1-TOM, topological overlap matrix), of all differentially expressed genes. The clustering tree shows the eight identified modules. **(C)** The module-trait relationships were evaluated by correlating module eigengenes with COVID-19 GI manifestations and immune cells. **(D)** Dendrogram of consensus module eigengenes obtained from WGCNA showing the consensus correlation. Correlations between the 7 modules are shown, where red indicates a strong connection and blue indicates the exact opposite.

Given the pivotal roles of macrophages in COVID-19 progression, we extracted gene signatures related to clinical features and immunological reactions from RNA-seq data for COVID-19 GI tissues. To construct a scale-free network, we set the soft threshold to 9 (*R* ^2^ = 0.86). Subsequently, we generated an adjacency matrix and a topological overlap matrix (Figure 4A). Using average hierarchical clustering and dynamic tree clipping, we identified eight modules (Figure 4B). For each module, we computed correlations between each eigengene and clinical features such as age, disease classification, diarrhea, nausea, vomiting, acid reflux, upper GI hemorrhage, hepatic function impairment, total bilirubin, glutamic pyruvic transaminase (ALT), glutamic oxaloacetic transaminase (AST), lymphocyte infiltration, plasma cell infiltration, neutrophil infiltration, interstitial vasodilation, interstitial edema, monocytes, macrophages, and CD4^+^ T-cells (Figure 4C). We observed that the MEgray module contained genes not belonging to any other module, while the MEgreen module exhibited significant connections with inflammatory cell infiltration (particularly macrophages, *R* = 0.84, *p* = 0.001; CD4^+^ T-cells, *R* = −0.62, *p* = 0.004) and other clinical features (Figure 4C). In contrast with MEgreen module, the Meblue module showed a negative correlation with macrophages (*R* = −0.57, *p* = 0.007) and a favorable correlation with CD4^+^ T-cells (*R* = 0.69, *p* = 0.002). Using hierarchical clustering, we evaluated the connectedness of eigengenes, revealing details about the connections between paired gene coexpression modules. The eight gene modules were categorized into two clusters based on similar gene expression patterns, and the two combinations (module 1: MEgreen, MEbrown, and MEred; module 2: MEyellow, MEblue, MEturquoise, and MEblack) each displayed a high level of interaction (Figure 4D). Thus, these results suggest that the recruitment and spatial distribution interaction between macrophages and CD4^+^ T-cells may be influenced by different signaling pathways.

### 3.5 Functional correlation analysis and protein—protein interaction (PPI) network construction

**FIGURE 5:**
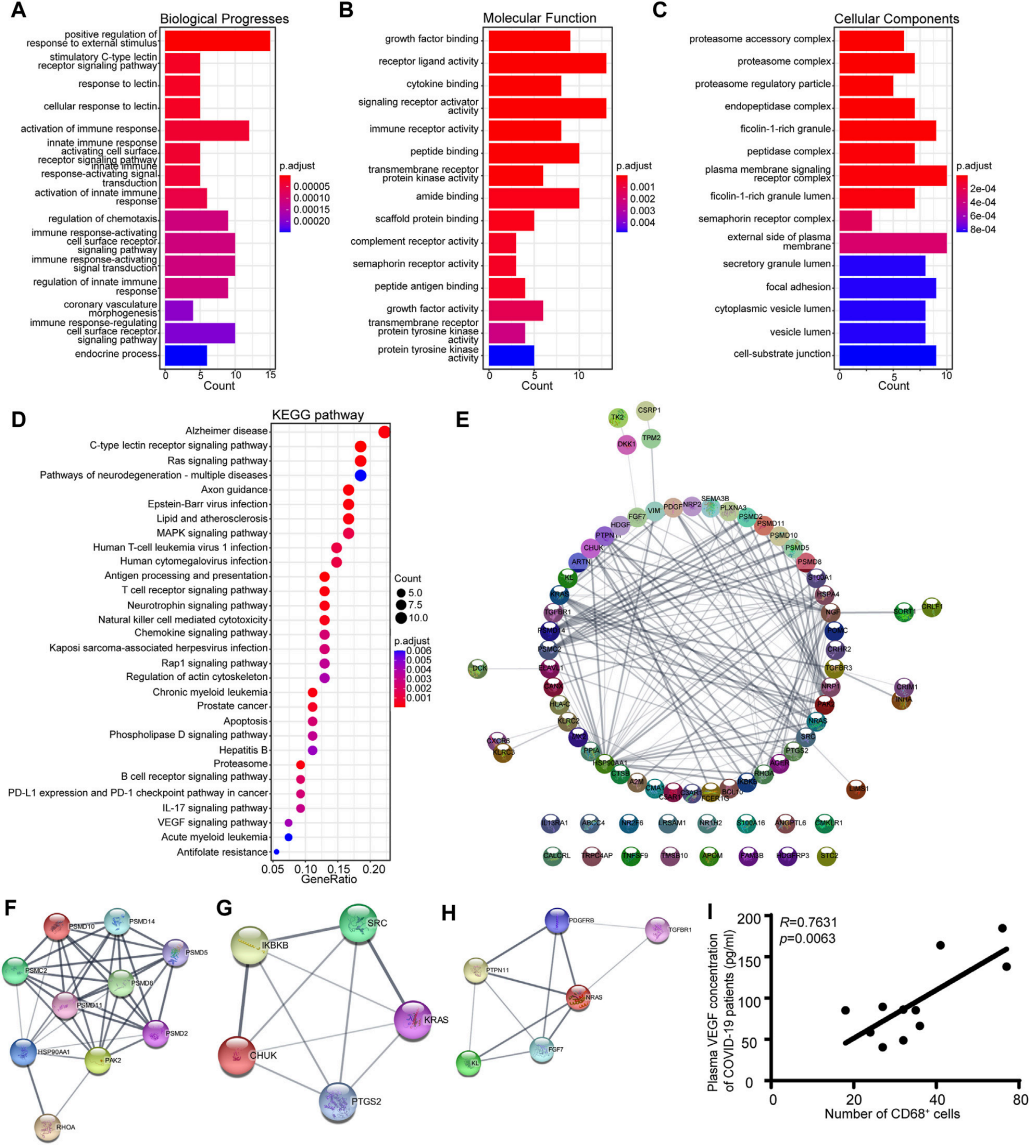
KEGG pathway analysis and GO functional enrichment analysis of the eigengene genes in the MEgreen module. **(A)** Biological process annotation diagram. **(B)** Molecular function annotation diagram. **(C)** Cellular component annotation diagram. **(D)** KEGG analysis of the MEgreen module. **(E)** A protein‒protein interaction network was constructed using Cytoscape software. The nodes represent proteins, and the edges represent their interactions. **(F)** Visualization of the hub genes involved in natural killer cell-mediated cytotoxicity. **(G)** Visualization of the hub genes of the chemokine signaling pathway. **(H)** Visualization of the hub genes of the VEGF signaling pathway. **(I)** Scatter plots showing a positive correlation between CD68^+^ macrophages and VEGF.

Gene Ontology (GO) and Kyoto Encyclopedia of Genes and Genomes (KEGG) pathway enrichment analyses were also conducted on the genes in the MEgreen module to identify potential biological processes associated with the inflammatory response in patients with COVID-19 (Kanehisa et al., 2008). The results demonstrated that the MEgreen module was linked to biological processes that might activate an immune response, including the innate immune response activating the cell surface receptor signaling pathway, immune response-activating signal transduction, and regulation of the innate immune response (Figure 5A). The molecular functions included receptor ligand activity, cytokine binding, immune receptor activity, and complement receptor activity (Figure 5B), while the cellular components included the proteasome accessory complex, plasma membrane signaling receptor complex, and cytoplasmic vesicle lumen (Figure 5C). KEGG pathway enrichment analysis also revealed that the MEgreen module was involved in the following signaling pathways: C-type lectin receptor, antigen processing and presentation, T-cell receptor, natural killer cell-mediated cytotoxicity, B-cell receptor, chemokine signaling pathway, and VEGF signaling pathway (Figure 5D). The abovementioned results suggested that the MEgreen module is closely related to the inflammatory response, including the activation of the immune response, the activation of the innate immune response, immune response-activating signal transduction, and the regulation of chemotaxis. For the hub genes in the MEgreen module, we constructed a PPI network using the STRING database, and we further identified three key gene subpathways and their relevant hub genes using Cytoscape’s MCODE algorithm (version 3.7.2) (Figure 5E–H). Finally, based on the MCODE scores, we observed enrichment of KEGG pathways related to natural killer cell-mediated cytotoxicity (PSMD10, PSMD14, PSMD5, PSMD8, PSMC2, PSMD11, PSMD2, HSP90AA1, PAK2, and RHOA), the chemokine signaling pathway (SRC, KRAS, CHUK, PTGS2, and IKBKB) and the VEGF signaling pathway (NRAS, PTPN11, KL, FGF7, PDGFRB, and TGFBR1) (Figure 5F–H). The expression level of each hub gene in patients is shown in Supplementary Table S1. Indeed, we further measured the plasma VEGF expression levels of COVID-19 patients and confirmed the favorable correlation between macrophages and VEGF (Figure 5I).

The MEred module, which was associated with the MEgreen module, was subjected to GO and KEGG pathway enrichment analyses to explore its biological functions in the inflammatory response in COVID-19 patients. Not surprisingly, genes involved in processes such as positive regulation of the response to external stimuli and activation or regulation of the innate immune response were enriched in biological processes (Supplementary Figure S3A). The molecular functions were associated predominantly with cytokine activity, cytokine receptor binding, and growth factor activity, while the cellular components were enriched in the proteasome regulatory particle, proteasome accessory complex, and immunoglobulin complex (Supplementary Figures S3B, C). The key immune-related pathways identified by KEGG pathway enrichment analysis included cytokine‒cytokine receptor interaction, natural killer cell-mediated cytotoxicity, the mitogen-activated protein kinase (MAPK) signaling pathway, and the chemokine signaling pathway (Supplementary Figure S3D). The pivotal genes in the MEred module were analyzed using a PPI network (Supplementary Figure S3E). Collectively, these data suggest that the MEgreen module is essential for the immune response to COVID-19.

## 4 Discussion

Angiotensin-converting enzyme 2 (ACE2) serves as the primary receptor for the cellular entry of SARS-CoV-2 and is widely distributed throughout the entire GI tract. Consequently, the GI system is susceptible to SARS-CoV-2 infection, leading to corresponding clinical symptoms (Elmunzer et al., 2023). Multiple studies have detected viral particles and RNA from SARS-CoV-2 in fecal samples, indicating the presence of the virus in the digestive system (Xiao et al., 2020). Moreover, emerging research has consistently reported a higher incidence of GI disorders, including irritable bowel syndrome, functional diarrhea, and fecal incontinence, in COVID-19 patients. However, the mechanisms underlying the GI functional abnormalities induced by SARS-CoV-2 infection have yet to be systematically elucidated.

Our previous research demonstrated that SARS-CoV-2 GI infection stimulates the production of cytokines, such as VEGF, which might contribute to systemic inflammation and be associated with disease progression (Wu et al., 2021; Zeng et al., 2022). The landscape of immune cells, such as those in the stomach, duodenum, and rectum, present in COVID-19 patients is unclear. Thus, in this study, we comprehensively explored GI tract-infiltrating immune cells in SARS-CoV-2 N protein-positive patient tissues (NP^+^ tissues) via IMC. A conspicuously expanded CD68^+^ macrophage population was found in these NP^+^ tissues. Specifically, a gene module related to CD68^+^ macrophages resulted in immune cell infiltration and activation. In COVID-19 patients, abnormal activation of macrophages expressing M1/M2 polarization markers (CD68, CD80, CD163, and CD206) in the lung is associated with the production of cytokines, including IL-6, IL-10, and TNF-α, as indicated by previous studies (Lian et al., 2022). Similarly, in the neural tissues of patients with severe COVID-19, the presence of CD68^+^ activated microglia and CD8^+^ cytotoxic T lymphocytes exacerbates the severity of neuroinflammation (Ruz-Caracuel et al., 2022). An examination of the infiltration of cardiac tissues also demonstrated significant infiltration of CD3^+^ and CD8^+^ cytotoxic lymphocytes, accompanied by the presence of CD68^+^ macrophages (Maiese et al., 2021). Thus, CD68^+^ cells, which are proinflammatory cells, were identified in our IMC results (Figure 1C; Figure 2D, Supplement Figures 1A–C) and are likely to be crucial cells contributing to GI inflammation and functional abnormalities.

In COVID-19, heightened inflammation in the GI tract, which is the body’s largest immune organ, is likely to contribute to disease progression, making it a key predictor of disease severity and morbidity. The NF-κB pathway is responsive to stress stimuli, such as cytokines, pathogenic infection, and environmental stress, and activates downstream effectors such as transcription factors to enhance the production of proinflammatory cytokines (Cuadrado and Nebreda, 2010). Additionally, evidence suggests that NF-κB plays a pivotal role in the pathogenesis of COVID-19. Aberrant NF-κB activation leads to increased numbers of immune cells and more intense cytokine storms, exacerbating extrapulmonary complications and systemic effects (Kesika et al., 2024; Zhou et al., 2024). SARS-CoV-2 NPs have been reported to promote the activation of the NF-κB signaling pathway by enhancing the association between TAK1 and the IKK complex. With viral RNA, the NP undergoes liquid-liquid phase separation (LLPS) to recruit TAK1 and the IKK complex, leading to the promotion of NF-κB activation. The CTD of the SARS-CoV-2 NP plays a critical role in its LLPS and NF-κB activation (Wang et al., 2022). Therefore, this finding also confirms that p-NFκB can be highly expressed in SARS-CoV-2 NP^+^ cells (Figure 2A).

To better understand the immune cell dynamics in the GI tract, we conducted a spatial neighbor analysis, comparing NP^−^ and NP^+^ samples per previous publications. Within the NP^+^ tissue cell clusters (Figure 3A–D), NP^+^ cells (cluster 11) notably surrounded CD8^+^ T-cells (cluster 4), CD4^+^ T-cells (cluster 5), CD20^+^ B-cells (cluster 13), and spatially exclusive interactions to CD68^+^ macrophages (cluster 9). Importantly, the interaction between CD68^+^ macrophages and cells with multiple phenotypes, especially CD4^+^ T-cells (cluster 5), was significantly decreased in NP^+^ tissue samples relative to NP^−^ tissue samples. These findings demonstrated extensive clustering of CD68^+^ immune cells in the NP^+^ group and weakened interactions between CD68^+^ cells and CD4^+^/CD8^+^ cells, changing the immunoreaction, as previously reported at neural and cardiac sites (Bearse et al., 2021; Colombo et al., 2021). Thus, the aggregation of CD68^+^ immune cells and their attenuated interaction with CD4^+^/CD8^+^ cells may be crucial factors leading to GI inflammation.

T-cells are essential for the immune system’s reaction to SARS-CoV-2 infection. Studies have confirmed that the subsequently produced CD8^+^ T-cells highly express NKG2A, which is regulated by the IL-6/STAT3 signaling pathway and induces CD8^+^ T-cell inactivation (Zheng et al., 2020). It has also been shown that activated CD4^+^ T-cells produce cytokines that further induce activation of other immune cells, especially CD8^+^ T-cells (Bhardwaj et al., 2022; Kawasaki et al., 2022). Naïve CD8^+^ T-cells undergo massive clonal expansion as they contact APCs and differentiate into effector T-cells to kill viruses (Bhardwaj et al., 2022). The interplay between macrophages and T-cells at the intestinal mucosal interface plays a key role in maintaining mucosal immune homeostasis (Mann and Li, 2014). Several studies have demonstrated that patients with severe COVID-19 have increased inflammatory monocyte and CD68^+^ macrophage infiltration and a corresponding decrease in anti-inflammatory alveolar macrophages (Wauters et al., 2021; Chen et al., 2022). Indeed, similar results were observed in SARS-CoV-2-infected digestive tract tissues in our study (Figure 3A–D). Recent evidence regarding the active role of intestinal epithelial cells (IECs) within the mucosal immune system has revealed that they act as nonprofessional APCs, activating subsets of T-cells with regulatory functions. IECs do not express costimulatory molecules such as conventional CD80 and CD86 but do express B7 family members, such as ICOS-L and PD-1L (Shao et al., 2005). Hence, under certain conditions, antigens presented by IECs suppress rather than increase the immune response (Nakazawa et al., 2004; Roda et al., 2010). The detailed mechanisms underlying this effect should be investigated in future studies.

To gain a deeper understanding of CD68^+^ macrophages and their interactions with CD4^+^ and CD8^+^ cells, we conducted RNA-seq analysis of COVID-19 GI tissues and WGCNA to identify key hub genes. Based on the WGCNA results, we identified a gene module predominantly associated with macrophages (MEgreen) and another module interacting with macrophages (MEred). The genes in the MEgreen module exhibited the strongest correlation with macrophage activation and were also associated with GI and hepatic dysfunction. Additionally, genes in this module were enriched in processes related to immune response activation, innate immune response activation, signal transduction of immune response activation, and regulation of chemotaxis. Indeed, macrophages can secrete chemokines to recruit immune cells or produce proinflammatory cytokines, resulting in cytokine storm-associated shock, multiple organ failure, and death in COVID-19 patients. Therefore, WGCNA of GI tissues from COVID-19 patients further highlighted the potential crucial role of CD68^+^ macrophages in the abnormal GI function observed in COVID-19 patients.

In our previous investigation, we discovered that the receptor-binding domain of the SARS-CoV-2 spike protein induces the overproduction of VEGF in enterocytes through the Ras-Raf-MEK-ERK pathway (Zeng et al., 2022). This phenomenon not only acts as a proinflammatory factor participating in the intestinal inflammatory response but also serves as a vascular permeability regulator, inducing local vascular leakage and the extravasation of inflammatory cytokines. In the present study, our analysis revealed that genes within the MEgreen module were significantly enriched in both the VEGF and MAPK signaling pathways. This finding reinforces the idea that the MEgreen module plays a crucial role in the immune response during COVID-19 in the GI tract. Further examination through GO and KEGG analyses of genes within the MEgreen module confirmed the enrichment of pathways related to VEGF and MAPK. The MAPK pathway, a key mediator of inflammation, is activated by various inputs, including the p38 MAP kinase pathway, Ras-Raf-MEK-ERK pathway, c-Jun kinase/stress-activated pathway, and MEK5/ERK5 signaling pathway. Elevated p38 MAPK activity, as implicated in our study, has been associated with adverse outcomes such as platelet aggregation, arterial thrombosis, endothelial cell apoptosis, hypoxia vasoconstriction, and vascular remodeling (Chen et al., 2018; Grimes and Grimes, 2020). Given the potential detrimental effects of COVID-19, targeting the MAPK pathway has been proposed as a viable treatment strategy for alleviating the cellular damage and adverse hyperinflammatory effects associated with this disease (Valipour et al., 2022). Thus, our findings underscore the pivotal role of the MEgreen module in the GI tract immune response during COVID-19, linking it to the dysregulation of VEGF expression and the activation of the MAPK pathway, providing insights into potential therapeutic approaches for addressing COVID-19-related complications.

Overall, we characterized macrophage activation as a featured immune response in SARS-CoV-2 infection of the GI tract and proposed a set of genes that are determinants of COVID-19 progression. Activation of the p38 MAPK pathway might be a key event during GI tract infection in patients with COVID-19. Therefore, our study provides new insight into the mechanism of the inflammatory response in acute COVID-19 syndrome and highlights potential therapeutic targets for the relief of GI symptoms in COVID-19 patients.

## Data Availability

The datasets presented in this study can be found in online repositories. The names of the repository/repositories and accession number(s) can be found in the article/Supplementary Material.
