# Supplementary material for: Spatial immune landscapes of SARS-CoV-2 gastrointestinal infection: macrophages contribute to local tissue inflammation and gastrointestinal symptoms
Source: Front Cell Dev Biol. 2024 Jul 17;12:1375354. doi: 10.3389/fcell.2024.1375354 (PMC11295004; doi:10.3389/fcell.2024.1375354)
Supplement: Supplementary file 1 [file DataSheet1.docx]

Supplementary Material

# Supplementary Tables

Supplementary Table 1. Key gene expression in the 3 key gene subpathways.

# Supplementary Figures


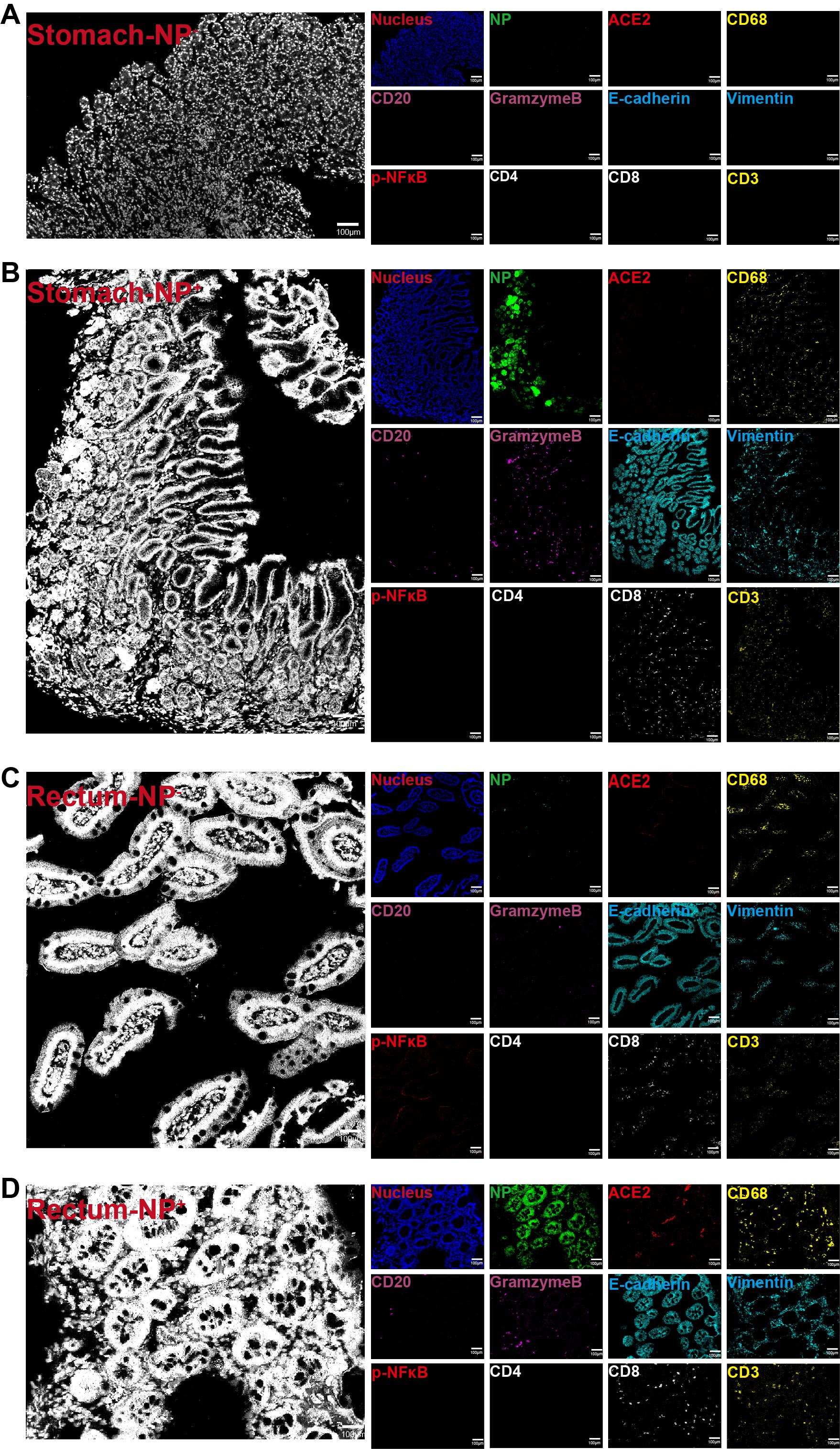


**Supplementary Fig. 1** Representative IMC images of the GI tract in patients with COVID-19. (A-D) Staining for NP, ACE2, CD68, CD20, Gramzyme B, E-cadherin, Vimentin, p-NFκB, CD4, CD8 and CD3 in NP-positive and NP-negative stomach and rectum tissue samples.


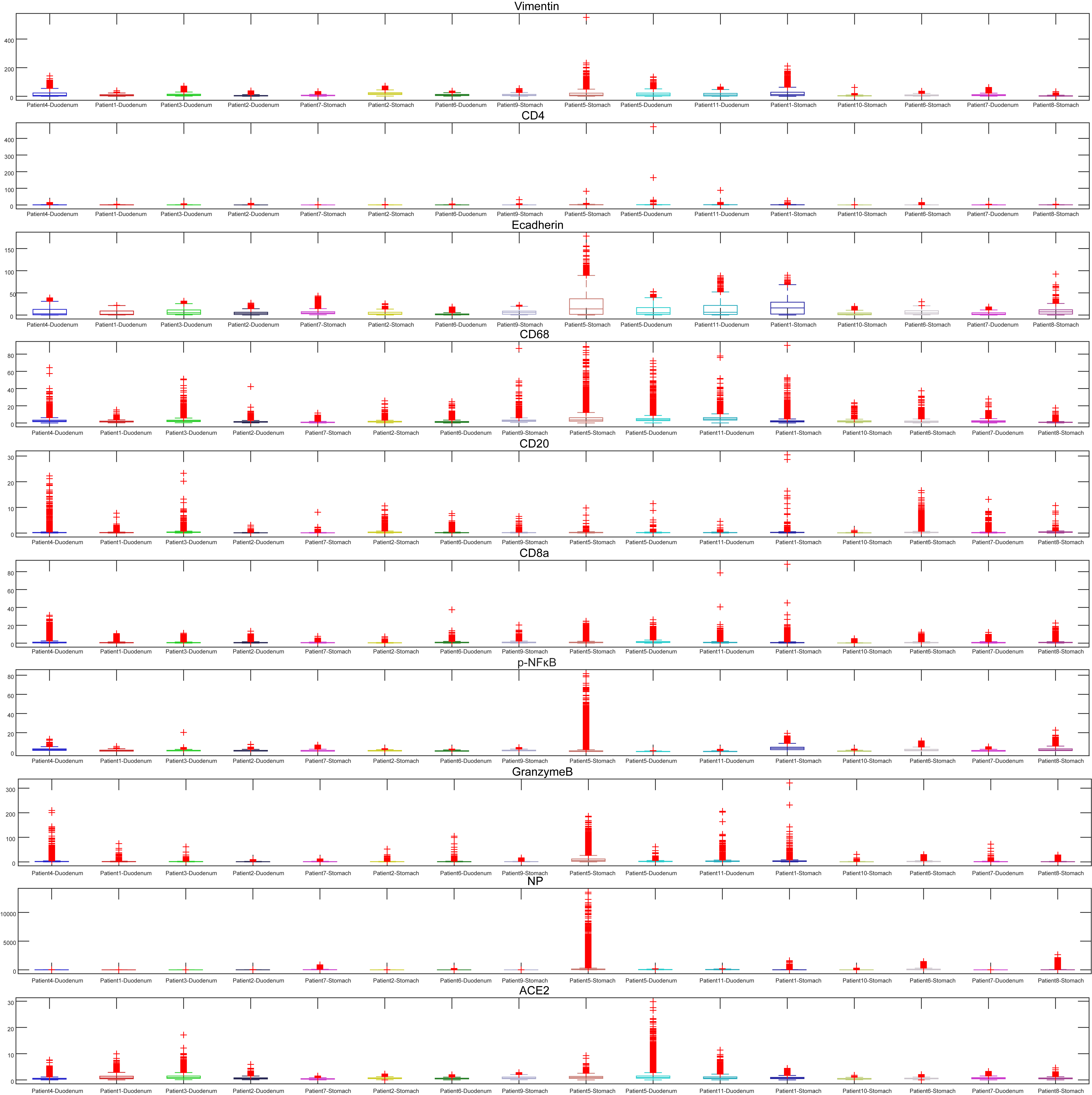


**Supplementary Fig. 2** Boxplots displaying the expression of several cell types, such as CD4, CD8, CD20, CD68, vimentin, NP, ACE2, Granzyme B, E-cadherin, and p-NFκB, for the individual COVID-19 patients.

**
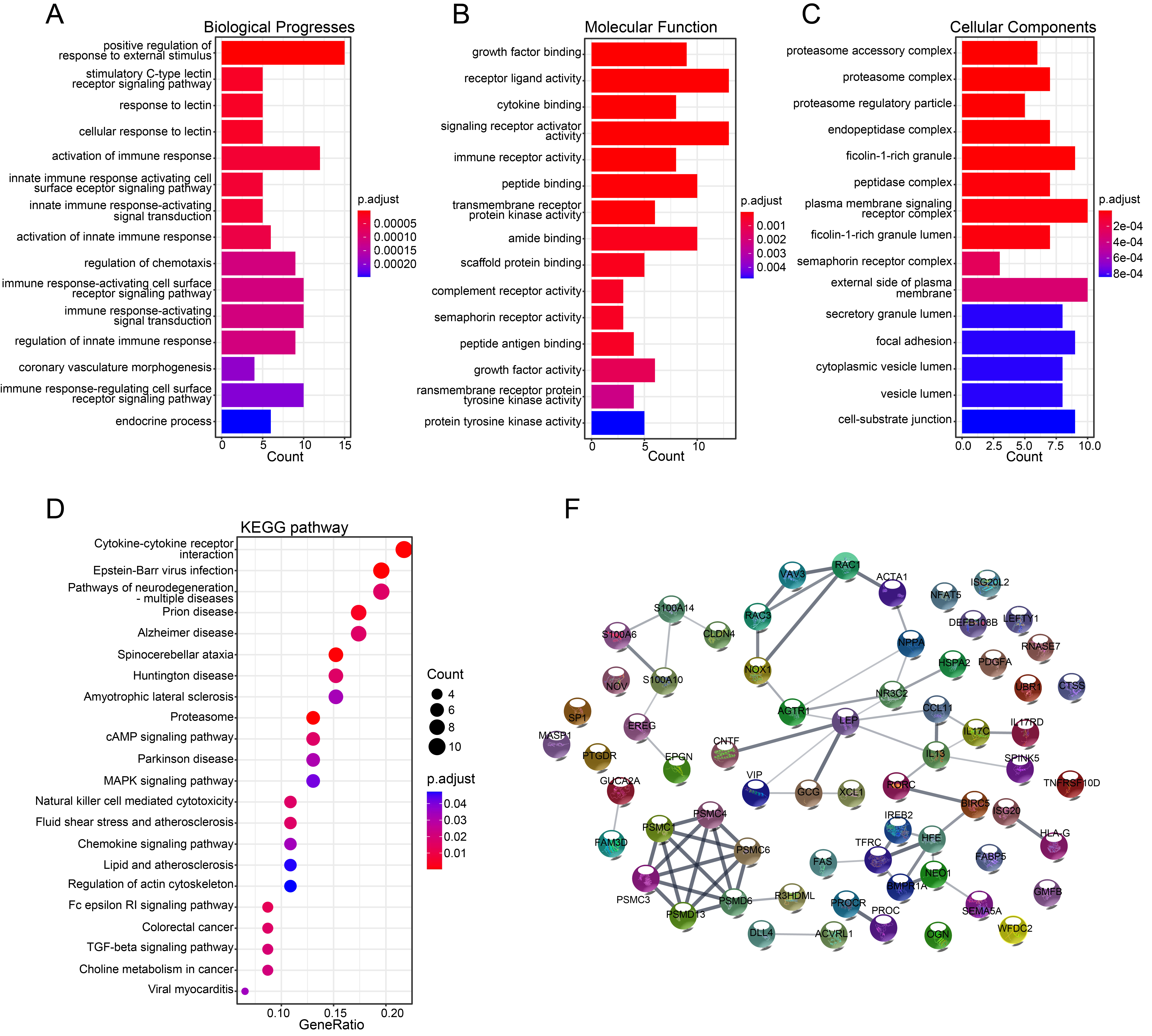
**

**Supplementary Fig. 3** KEGG pathway and GO functional enrichment analysis of the Mered module genes. (A) Biological process annotation diagram. (B) Molecular function annotation diagram. (C) Cellular component annotation diagram. (D) KEGG annotation diagram. (E) PPI network construction.
